# How to Get All the Money Required

**Published:** 1919-11-29

**Authors:** 


The Hospital

The Workers' Newspaper of Administrative Medicine and Institutional Life, Administration, National Insurance and Health.

No. 1747, Vol. LXVII. SATURDAY, NOVEMBER 29, 1919. PRICE TWOPENCE

### HOW TO GET ALL THE MONEY REQUIRED.

It seems to us that neither the hospitals nor the newspapers have yet come to appreciate fully the position in which voluntary hospital finance is placed to-day after the war. It is true that, looked at in one way, the impediments to the raising of funds, the special circumstances which tend at first sight to increase the difficulty of securing an adequate income for our hospitals, are serious. Amongst them is the heavy expenditure entailed upon most people through the war and the existing conditions of to-day. These impediments have appeared insurmountable by the routine raiser of money in the past. But our view is that, rightly understood and squarely met, they will under the guidance of a zealous, able, and experienced man of business, who has his heart in the work, prove stimulating conditions to the joy to be won by success, when fighting in the cause of the sick in the days immediately following the cessation of war. What, then, is the position, and what must it entail if an adequate income is to come to the voluntary hospital? First, that the people of all classes throughout the nation, as the Two-Minutes' Silence proved, have their hearts not only with those who have made the supreme sacrifice, but with the sick and suffering, to an extent probably never before so manifest in the history of the nation. To have lost one's nearest and dearest, to encounter the vacant chair in the family circle, to remember all the joys of companionship and association and the severity of the grief caused initially and continuously, all tend to excite a desire in the best of us in the days of health to render some service, personal, direct or indirect, to those who suffer and claim our thoughts, our service, and our monetary help for the institution without which their lot would be grievous indeed. Here we have new sources, new fields to cultivate, new forms of approach to find and practise, and an accretion of a multitude of willing hearts and service which will come to every hospital that has in charge of its business in these times a man or woman who has the heart, the brains, the courage, and the will to enter the service of the sick. To do so, is to win consolation for oneself, and splendid help for our hospitals.

Again in the course of years Mr. Chance, of Birmingham, and able men in other places, have made it their business to ascertain with infinite labour and care the number of people who, out of a population of a great city in these islands, give anything at all each year in support of the hospitals. This service—for it was a most substantial service rendered by the more intelligent of hospital supporters in these islands—should be taken to heart in these days by the faint hearts who confine their attention to the lists they have, in connection with their institution, of persons from whom most if not all the money received has come in recent years. They forget too often that when the total is forthcoming it usually does not represent a tithe of the population, or even probably of the people who have money to spare, and only have lacked the will to give. Before the war we have had many instances in the course of our life of people, wealthy, able, surrounded by every comfort that money can bring, who lacked the power to give substantial sums in their lifetime. We have even known cases where an appeal after dinner by one of their own immediate friends who had learnt the joy of giving to approved objects in his lifetime, has produced a tentative promise to give a cheque in the morning. After breakfast the relaxed grip of the evening failed, with the confession " I cannot give except by will." This was no isolated experience but represents the unfortunate position of many people who are to be pitied for their failure to realise how to enjoy life and see the best of days. We believe that this state of mind in very many cases must have been changed by the war, and a zealous and able guardian of the hospital's privy purse will welcome the opportunity that the present stress gives him to try these golden pheasants of humanity in their new attitude of mind created by the experiences, the trials, anxieties, and sufferings of war-time. Most of them know, no doubt, the awakened interest and the opening to new sources of joy in the pleasures of living and taking their part in the endeavour to leave the world a little better than they found it.

Then there is the fact which so many of the croakers seem to forget, that, if, as must have happened, some or even most of the hospital's supporters are under the harrow in a monetary sense, to a greater or less extent, there are in most centres of population throughout these islands a surprisingly large number of persons who have accumulated wealth out of the war, or whose monetary resources have been largely increased and continue to increase at the present time. They no doubt would include very many of the nine-tenths of the population who heretofore have not troubled about Lazarus or his needs. Here is a new field offering a splendid harvest to the capable, the faithful, the resourceful and the true-hearted of both sexes, who believe in rendering personal service in the cause of the sick. Why, the writer feels young again as he writes these words, because of the glory of the opportunity and the assured certainty of the success which lies before the reaper who is faithful to his beliefs, and zealous and unselfish in the work he puts into the business of money-raising for our splendid hospitals throughout the country. We have not space to follow out in detail the many new sources that are open to cultivation where the needs of the sick, the claims of the sick, make their presence understood and felt by all classes of the community. We make bold to think and to say that in fact the few millions heretofore given to hospitals by some tithe of the population of these islands can be doubled or trebled, if need be, providing steps are taken by zealous men and women to organise the effort in every centre which has the blessing of a hospital ministering continually to the needs of its population.

Such thoughts as these, tested and found sound and life-giving as they will be to the true-hearted, will bring a new and continuous flow of life into a community which is fortunate enough to recruit for its hospitals a small army of able workers under a wise and foreseeing keeper of its hospital's privy purse. To sit and utter the words: " We must secure State Aid! " Shades of our forbears and sires! What are we coming to, we English people, who have got down so low as to disregard the ancient teaching which has made England what she is to-day? What is that teaching? God helps those who help themselves. The State has more than enough to do to mop up the mess in all directions arising out of the late terrible war. Why, the State cannot thrive if the English people, much less the hospitals, and those to whose zealous labour in the past we owe freedom, cry out and worry the Government for money they have not got to give, when all the time the money is around the tired and worn veteran, who can find immediate relief and take his well-earned rest by finding a live man or woman, men or women, to give some of the extra hours of leisure which are now coming to most people, to do their part in the days of health in the cause of the sick. We are happy to have the assurance which is coming in by the post with encouraging freshness, that the policy of taking the present hospital crisis lying down is regarded by an increasing number of British people as positively degrading and impossible for members of the British race. More who are young and active enough and intelligent enough to look all round a great question like this, soon resolve to do their part to the utmost to right the good ship "Hospital," and bring her safely into port, more heavily laden with the means she urgently requires to do everything that is humanly possible to help the sick in all classes by the best methods, and in the quickest possible time, to the recovered health which will bring wealth to the nation and joy to the home.

**The previous article appeared on November 22, p. 155.**

